# Practice patterns and adherence to society guidelines for suspected choledocholithiasis: A comparison of academic and community settings from a large US healthcare system

**DOI:** 10.3389/fmed.2022.1000368

**Published:** 2022-10-20

**Authors:** Shahrooz Rashtak, Hemant Goyal, Aswathi Chandran, Bhavtosh Dedania, Prithvi Patil, Vaibhav Wadhwa, Sushovan Guha, Tomas Davee, Srinivas Ramireddy, Nirav Thosani

**Affiliations:** Center for Interventional Gastroenterology at UTHealth (iGUT), McGovern Medical School, University of Texas Health Science Center, Houston, TX, United States

**Keywords:** choledocholithiasis, practice guidelines, adherence, American Society of Gastrointestinal Endoscopy (ASGE), endoscopic retrograde cholangiopancreatography (ERCP)

## Abstract

**Background:**

The American Society of Gastrointestinal Endoscopy (ASGE) has proposed practice guidelines for evaluating patients with suspected choledocholithiasis. This study aims to assess and compare practice patterns for following ASGE guidelines for choledocholithiasis in a large academic vs. community hospital setting.

**Methods:**

A total of one thousand ER indicated for choledocholithiasis were randomly selected. Patients’ demographics, total bilirubin, imaging studies including magnetic resonance cholangiopancreatography (MRCP), intraoperative cholangiogram (IOC), endoscopic ultrasound (EUS), and ERCP results were retrospectively collected. Patients with prior sphincterotomy were excluded. We examined the following practice deviations from the current ASGE guidelines; (1) ERCP was potentially delayed in high probability cases while awaiting additional imaging studies, (2) ERCP was performed without additional imaging studies in cases of low/intermediate-risk, or (3) ERCP was performed in low/intermediate-risk cases when additional imaging studies were negative.

**Results:**

A total of 640 patients with native papilla who underwent ERCP were included in the final analysis. Overall, the management of 43% (275) of patients was deviated from the applicable ASGE guidelines. Academic and community provider rates of non-adherence were 32 vs. 45%, respectively (*p*-value: < 0.01). Of 381 high-risk cases, 54.1% had additional imaging before ERCP. (Academic vs. community; 11.7 vs. 88.3%, *p*-value: < 0.01). In 26.7% (69/258) of low/intermediate risk cases, ERCP was performed without additional studies; academic (14.5%) vs. community (85.5%) (*p*-value: < 0.01). Finally, in 11.2% (19/170) of patients, ERCP was performed despite intermediate/low probability and negative imaging; academic (26.3%) vs. community (73.7%) (*p*-value: 0.02).

**Conclusion:**

Our study results show that providers do not adhere to ASGE practice guidelines in 43% of suspected choledocholithiasis cases. The rate of non-adherence was significantly higher in community settings. It could be due to various reasons, including lack/delays for alternate studies (i.e., MRCP, EUS), concern regarding the length of stay, patient preference, or lack of awareness/understanding of the guidelines. Increased availability of alternate imaging and educational strategies may be needed to increase the adoption of practice guidelines across academic and community settings to improve patient outcomes and save healthcare dollars.

## Introduction

Practice guidelines are developed by reviewing relevant literature and incorporating expert opinions to provide evidence-based recommendations to aid clinicians with the decision-making and management of a specific condition. Adherence to the relevant society guidelines has been shown to reduce variations in clinical practice and improve patient outcomes (1–3). Therefore, authorities, regulatory agencies, and payers often consider compliance with guidelines the “standard of care,” and healthcare practitioners (HCPs) are expected to follow society’s recommendations. Adherence to guidelines can vary among clinicians and is sometimes poorly practiced in certain settings (4, 5). These guidelines are to “guide” the HCPs to treat patients in appropriate clinical scenarios, and deviation can occur on a case-by-case basis, but there are other factors related to non-adherence (6).

**FIGURE 1:**
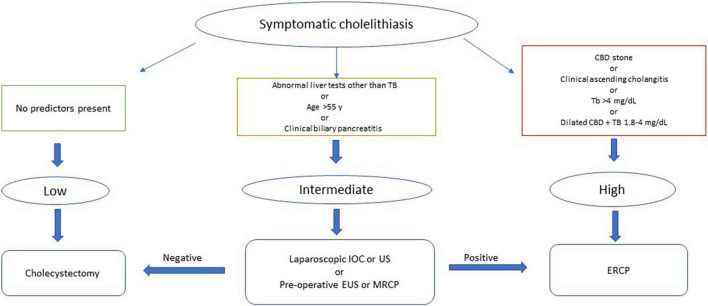
American Society of Gastrointestinal Endoscopy 2010 guidelines for the management of patients with symptomatic choledocholithiasis. TB, Total bilirubin; CBD, Common bile duct; IOC, Intraoperative cholangiogram; US, Ultrasound; EUS, Endoscopic ultrasound; MRCP, Magnetic resonance cholangiopancreatography; ERCP, Endoscopic retrograde cholangiopancreatography.

Choledocholithiasis (CDL) is commonly managed by endoscopic retrograde cholangiopancreatography (ERCP). In the last two decades, ERCP has evolved from a diagnostic modality to primarily a therapeutic intervention with advancements in non-invasive imaging techniques. However, ERCP can be life-saving in septic patients due to ascending cholangitis but can be associated with complications including acute pancreatitis etc. in 6–15%, and prolonged hospitalizations and death in 1–2% of cases (7, 8). The American Society for Gastrointestinal Endoscopy (ASGE) published practice guidelines for the management of suspected CDL in 2010 (9), which was revised in 2019 to increase specificity and the positive predictive value (PPV) of predicting the presence of bile duct stones (10). According to 2010 criteria, ERCP was recommended without the need for non-invasive studies in high-risk patients, defined as one of the following clinical characteristics: (1) ascending cholangitis, (2) CDL on imaging, (3) total bilirubin (TB) > 4 mg/dl or (4) TB between 1.8 and 4 mg/dl and dilated common bile duct (CBD) on imaging. For those at intermediate risk for CDL, which includes other abnormal liver biochemical tests, gallstone pancreatitis, age >55 years, or CBD dilation (without TB > 1.8 mg/dl), guidelines recommend using less invasive tests like endoscopic ultrasound (EUS), magnetic resonance cholangiopancreatography (MRCP) or intraoperative cholangiogram (IOC) during cholecystectomy. These tests have a diagnostic performance comparable to ERCP with a lower risk of adverse events (11, 12). Finally, laparoscopic cholecystectomy without bile duct imaging is recommended for patients with symptomatic cholelithiasis without any of the predictors (Figure 1).

These criteria are widely used by practicing gastroenterologists for risk assessment of CDL; however, the rate of adherence to these recommendations in different practice settings is unknown. In this study, we aim to evaluate how commonly clinical practice deviated from the guidelines and to compare practice patterns in academic vs. community hospitals.

## Materials and methods

In our hospital system, over 10,000 ERCPs were performed from 2013 to 2019, which are included in a prospectively maintained internal electronic database. We randomly selected 1,000 ERCPs performed for an indication of CDL. Data on demographics, hospital settings (academic or community), TB on initial presentation, CBD diameter on initial abdominal ultrasound or CT scan, and presence of CDL on imaging were collected by retrospective chart review. Additional information was collected regarding alternative tests like MRCP, EUS, or IOC and whether choledocholithiasis was detected; the presence of clinical ascending cholangitis; gallstone pancreatitis; and ERCP findings. For patients who underwent multiple ERCPs for CDL, only the index presentation and first ERCP findings were included. Patients with prior biliary sphincterotomy, history of biliary stricture, primary sclerosing cholangitis, history of chronic liver disease with baseline abnormal liver function test, and those without available baseline labs and initial imaging were excluded. This study was approved by the Institutional Review Board at UTHealth-Houston.

**FIGURE 2:**
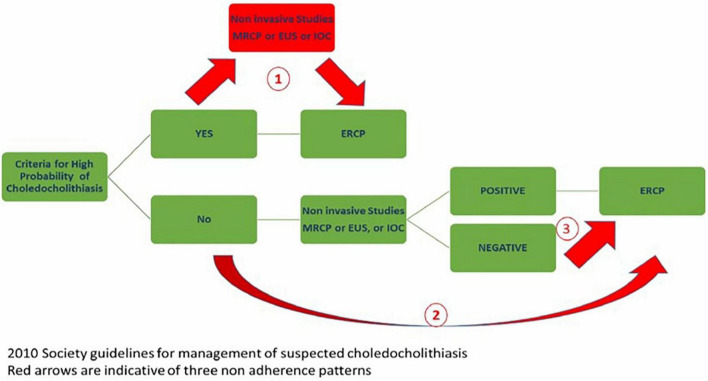
Choledocholithiasis management algorithm based on the 2010 ASGE practice guidelines with three non-adherence pathways causing delay in care, potential harm, and significant harm.

Based on initial laboratory data and imaging findings, each patient was categorized as low/intermediate probability or high CDL probability per the 2010 ASGE guideline. Dilated CBD was defined as CBD diameter >6 mm with an intact gallbladder or >8 mm in those with prior cholecystectomy (13). We defined clinical cholangitis as the presence of Charcot’s triad of abdominal pain, fever and/or leukocytosis, and abnormal liver test results on presentation. Subsequently, we examined how often practice deviations from 2010 guidelines occurred (Figure 2):

1. **Potential delay in care:** ERCP was potentially delayed awaiting additional imaging studies in high probability cases.
2. **Potential Harm:** ERCP was performed without additional imaging studies in cases of low/intermediate-risk.
3. **Significant Harm:** ERCP was performed in low/intermediate-risk cases when additional imaging studies were negative.

## Results

After reviewing the initial 1000 ERCP patients, a total of 640 records, academics vs. community; 107 (17%) vs. 533 (83%) patients with native papilla who underwent ERCP were included in the final analysis. The mean age of the entire cohort was 47.2 years; 44.6 vs. 47.7 years in academics vs. community, respectively. The gender ratio was also similar in the two groups, with 74.7% females; (academics vs. community 73.8% vs. 74.9%). There were no significant differences in the mean TB 2.8 (2.4 vs. 2.9) and mean CBD diameter of 8.3 mm (8.5 vs. 8.3) academics vs. community group, respectively (Table 1).

**TABLE 1:** Demographics and clinical characteristics comparing subjects in the academic vs. community practice groups.

		Overall (*n* = 640)	Academic (*n* = 107)	Community (*n* = 533)
Age		47.2 + 21.6 (11–96)	44.6 + 22.3 (11–94)	47.7 + 21.5 (17–96)
Gender	Female	476 (74.7%)	78 (73.6%)	398 (74.9%)
	Male	161 (25.3%)	28 (26.4%)	133 (25.1%)
Choledocholithiasis on initial imaging	Yes	115 (18%)	16 (15%)	99 (18.6%)
	No	524 (82%)	91 (85%)	433 (81.4%)
Total bilirubin		2.8 + 2.63 (0.1–35.4)	2.4 + 1.97 (0.2–9.2)	2.9 + 2.74 (0.1–35.4)
CBD Diameter (mm)		8.3 + 3.65 (1.3–24)	8.5 + 3.72 (2.4–19)	8.3 + 3.64 (1.3–24)

A total of 355 patients underwent alternative imaging studies prior to ERCP, among which 303 (85.3%) had positive results for CDL. Two hundred fifty patients underwent MRCP, of which 205 (82%) had positive results. Fifty-nine intraoperative cholangiograms (IOC) were performed during cholecystectomies, of which 57 (96.6%) were positive. Nineteen patients had a EUS, of which 18 (94.7%) were indicative of CBD stone or sludge.

Overall, deviation from the applicable ASGE guidelines was observed in 43% (275) of cases. The rate of non-adherence was 32 vs. 45% among academic and community physicians (*p*-value: < 0.01) (Table 2).

**TABLE 2:** Frequency of guidelines non-adherence causing delay in care, potential harm and significant harm comparing the academic vs. community setting.

	Academic	Private	*P*-value
Distribution of ERCP	107 (16.7%)	533 (83.3%)	
Potential delay (*n* = 206)	24 (11.7%)	182 (88.3%)	<0.01
Potential harm (*n* = 69)	10 (14.5%)	59 (85.5%)	<0.01
Significant harm (*n* = 19)	5 (26.3%)	14 (73.7%)	0.02

1. Potential delay in the standard of care: Of 381 high-risk cases, 54.1% (206/381) had additional imaging before ERCP; community vs. academics (88.3 vs. 11.7%, *p*-value: < 0.01).
2. Potential Harm: In 26.7% (69/258) of low/intermediate risk cases, ERCP was performed without additional studies; community vs. academic practice (85.5 vs. 14.5%, *p*-value: < 0.01).
3. Significant Harm: In 11.2% (19/170) of patients, ERCP was performed despite intermediate/low probability and additional negative imaging; community vs. academic practice (73.7 vs. 26.3%, *p*-value: 0.02).

## Discussion

The results of our study show that the ASGE practice guidelines for managing suspected CDL were not followed in about half of the cases. The guideline non-adherence was significantly higher in the community practice compared to the academic setting. These results are consistent with prior studies on failing to adhere to the North American and European guidelines. A study across eight universities of Toronto affiliated hospitals for management of gallstone pancreatitis showed that of 52 patients with image-confirmed CBD obstruction, only 16 (31%) underwent ERCP, with an average of 3.1 days after admission (14). Similarly, another study from the United Kingdom revealed that only one-third (32.1%) of patients with mild gallstone pancreatitis were managed as per British Society of Gastroenterology guidelines and underwent cholecystectomy during or within 2 weeks of the index admission. About 20% of the patients suffered further morbidity as a result of a delayed operation (15).

Guidelines are written to provide evidence-based recommendations to minimize variability in clinical practice and improve patient outcomes. Nevertheless, the circumstances for deviation from the guidelines are still unclear, and information about the potential barriers to guideline adherence is unavailable. However, these guidelines are not “rules or mandates,” and clinical decisions in certain cases are based on the patient’s condition and available resources. Therefore, the clinical situation of a given patient may lead an HCP to take a deviated course of action from guidelines. These guidelines are often applied while considering each unique patient’s social and ethical aspects and incorporating patient and family wishes in shared decision-making for managing a particular condition. The HCPs need to ensure and document that their recommendations are justifiably in the patient’s best interest, not driven by bias or conflict of interest. Clinicians are obligated to respect patient autonomy and clearly communicate the information about risks, benefits, and alternatives of available treatment options (16).

The potential barriers to guideline adherence are divided into guideline-related and clinician-related factors. Guideline-related factors include the complexity of the recommendation(s), multiple rules in a single guideline, the discrepancy between guidelines from different societies on a single disease, the perception that a guideline is outdated, and the lack of applicability of guidelines in general and specifically to individual patients. Clinician-related factors include incompetency and knowledge gaps in complex cases, unawareness of the most recent guidelines, overconfidence, time pressures, resistance to changing usual practice, and fragmentation of care (6, 17).

The findings of our study regarding the significant rate of non-adherence could be due to the patient and/or provider preference, as well as the availability of local resources. The difference in the academic and community setting could be partly explained by clinician-related barriers such as lack of readily available alternative studies (i.e., MRCP, EUS, or IOC), provider concern regarding the length of stay, or lack of understanding of the guidelines. Participation in scientific meetings, such as multidisciplinary discussions, grand rounds, journal clubs, etc., in the academic setting may play a role in a better understanding and interpretation of recommendations, especially in complex cases. Additionally, the educational environment and assistance provided by the trainees in patient care would ease the time pressure that may otherwise affect clinicians in community settings. Also, the hierarchical and dynamic nature of the academic setting may further facilitate changing from routine practice. Although the factors mentioned above could potentially explain our findings, our study is limited in identifying the very specific barriers that further affect adherence in the private setting. The lack of available data about patient outcomes, especially in the non-adherent group, is another potential limitation of our study. Further studies are needed to directly compare the benefits of guideline adherence in patients with choledocholithiasis.

## Conclusion

In conclusion, ASGE guidelines for CDL management are not consistently followed among physicians from community and academic settings. However, non-adherence is more common with HCPs in the community setting. It could be related to a variety of factors, including clinician-related factors or limitations of the guidelines, such as relatively poor specificity and predictive value for the presence of bile duct stones. These results highlight the significance of increased awareness and further education about the guideline availability for CDL among HCPs, especially in the community setting.

## Data availability statement

The raw data supporting the conclusions of this article will be made available by the authors, without undue reservation.

## Ethics statement

The studies involving human participants were reviewed and approved by the Institutional Review Board at UTHealth—Houston. Written informed consent for participation was not required for this study in accordance with the national legislation and the institutional requirements.

## Author contributions

ShR, HG, and NT: conception and design. PP, AC, and BD: literature review. ShR: first draft. All authors: critical revision, editing and final approval.
